# On the use of plume models to estimate the flux in volcanic gas plumes

**DOI:** 10.1038/s41467-021-22159-3

**Published:** 2021-05-11

**Authors:** Julia Woitischek, Nicola Mingotti, Marie Edmonds, Andrew W. Woods

**Affiliations:** 1 BP Institute for Multiphase Flow, University of Cambridge, Cambridge, UK; 2 Department of Earth Sciences, University of Cambridge, Cambridge, UK

**Keywords:** Geophysics, Volcanology

## Abstract

Many of the standard volcanic gas flux measurement approaches involve absorption spectroscopy in combination with wind speed measurements. Here, we present a new method using video images of volcanic plumes to measure the speed of convective structures combined with classical plume theory to estimate volcanic fluxes. We apply the method to a nearly vertical gas plume at Villarrica Volcano, Chile, and a wind-blown gas plume at Mount Etna, Italy. Our estimates of the gas fluxes are consistent in magnitude with previous reported fluxes obtained by spectroscopy and electrochemical sensors for these volcanoes. Compared to conventional gas flux measurement techniques focusing on SO_2_, our new model also has the potential to be used for sulfur-poor plumes in hydrothermal systems because it estimates the H_2_O flux.

## Abstract (web summary)

Monitoring the flux of gas from volcanoes is a fundamental component of volcano monitoring programs and is used as a basis for eruption forecasting. Here, the authors present a new method using video images of volcanic gas plumes to measure the speed of convective structures and to estimate volcanic fluxes.

## Introduction

**Fig. 1:**
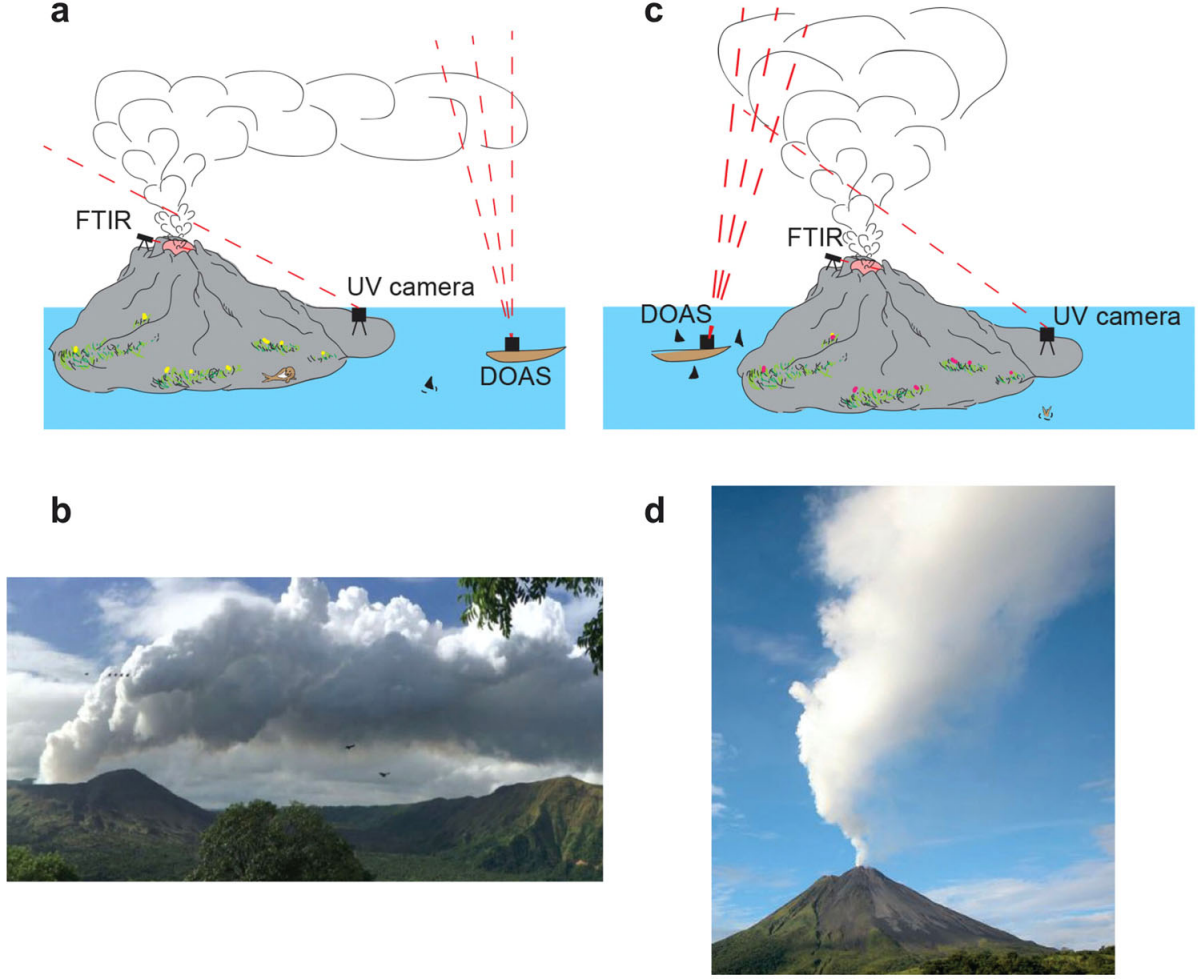
Volcanic plumes. The methods by which volcanic gases are typically measured include Fourier transform infrared spectroscopy (FTIR), Ultraviolet camera (UV camera) and Differential Optical Absorption Spectroscopy (DOAS). **a** Measurements in case of a wind-blown plume such as **b** the one observed at Masaya Volcano in Nicaragua. **c** Measurements in case of a vertical plume such as **d** the one observed at Arenal Volcano in Costa Rica. Photocredit: **b** obtained from vumo.cloud; **d** obtained from govisitcostarica.com.

Volcanic gas plumes are composed of water vapour, CO_2_, SO_2_ and a range of other gases, with the dominant gas typically being H_2_O (70–99%). The volcanic gas typically forms a buoyant plume, which is either carried downwind (Fig. 1a, b) or rises vertically (Fig. 1c, d) into the atmosphere.

A number of different approaches exist to measure the gas flux issuing from the volcano, many of which involve absorption spectroscopy in the mid-infrared or ultraviolet region, whereby the gas column amounts from a specific plume cross-section are integrated and recorded in time^1–5^. The flux of the gas can be then calculated by combining the mass in the cross-section with the plume speed (often the wind speed). The measurement of volcanic fluxes has largely focused on SO_2_ because of its low ambient concentrations and strong absorption signature in the UV region^6,7^, which make it easy to measure by remote sensing techniques^8^. The wind speed is estimated using the closest meteorological station^9,10^; alternatively, an anemometer can be used on the volcanic crater rim to measure the wind directly^11^. Another approach to measure the gas flux issuing from a volcanic vent is the cross-correlation method, which is used to estimate the mean speed of the plume by tracking features of the plume^5,12^ or by motion-tracking algorithms^13–15^. The SO_2_ flux data can be combined with data on the concentration of different volatile species measured in the vent of the volcano using integrated gas sensors^16,17^ (MultiGAS) or Fourier transform infrared spectroscopy (FTIR) instruments^18,19^ (see Fig. 1), to estimate the flux of other gases issuing from the volcano.

In this paper, we take a complementary approach to calculate gas fluxes by using models of turbulent buoyant plumes to analyse video records of a nearly vertical gas plume at Villarrica Volcano, Chile, taken in 2012^20^ and a wind-blown gas plume at Mount Etna, Italy, taken in 2015^21^. In particular, we describe the speed of the intermittent convective structures in these plumes as a function of the distance from the vent. We show that these measurements are consistent with classical plume theory for both vertical and wind-blown plumes. We then combine these measurements with some analogue laboratory experiments and historical plume data, to show how these measurements can provide an estimate of the gas flux in the plume. We compare these estimates with published measurements of typical gas fluxes at these volcanoes, and find they are of the same magnitude.

## Results

### Vertical plumes: laboratory experiments

In the case of a very low wind speed compared to the speed of the plume, a source of hot buoyant gas will lead to a near-vertical plume. In this situation, provided the ambient is unstratified, the classical theory of turbulent buoyant plumes^22^ suggests that the vertical speed, *u*, decreases with height, *z*, according to the law

1 $$u \sim \beta {B}^{\frac{1}{3}}{\left(z+{z}_{0}\right)}^{-\frac{1}{3}}$$

where *β* is a constant, *B* is the buoyancy flux of the plume, and *z*_0_ is the virtual origin of the plume, corresponding to the distance behind the actual source at which a point source with zero mass flux would produce the same plume^22,23^. We expect turbulent structures in the plume to move with speed proportional to *u*. This leads to the expression for the height of a turbulent structure as a function of time

2 $${\left(z+{z}_{0}\right)}^{\frac{4}{3}}=\alpha {B}^{\frac{1}{3}}\left(t-{t}_{1}\right)+{\left({z}_{1}+{z}_{0}\right)}^{\frac{4}{3}}$$

where *t* is time, *z*_1_ is the initial position of the structure at time *t*_1_ and *α* is a constant.

**Fig. 2:**
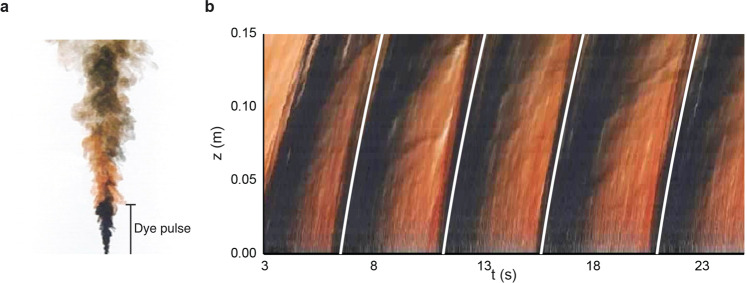
Laboratory vertical plumes. **a** Photograph from an experiment of a turbulent saline plume in fresh water. **b** Time series of a vertical line through the centre of the plume, in which a series of pulses of dye were added to the plume. The white lines track the leading edge of each dye pulse as it rises through the plume, and these lines are given by Eq. (2).

Bhamidipati and Woods^24^ reported experiments of a turbulent plume produced by the continuous release of salt water at the top of a tank filled with fresh water. By periodically injecting pulses of black dye into the saline source fluid and recording the descent of the dye front through the plume, they measured the speed of some of the turbulent structures in the plume. Figure 2 shows (a) a photograph of the plume and (b) a time series of a vertical line of pixels through the centre of the plume obtained from a video recording of the plume. There is a series of successive fronts between the black and the orange dyed fluid. By fitting the model Eq. (2) to these dye fronts, we estimate that

3 $$\alpha =5.6\pm 0.2$$

as may seen by the white lines in Fig. 2b, which have been plotted using Eq. (2) and the value of *α* estimated in Eq. (3). We note that this result is complementary to estimates of the speed of the edge of a vertical turbulent plume as reported by Burridge et al.^25^.

### Vertical plumes: Villarrica plume (2012)

**Fig. 3:**
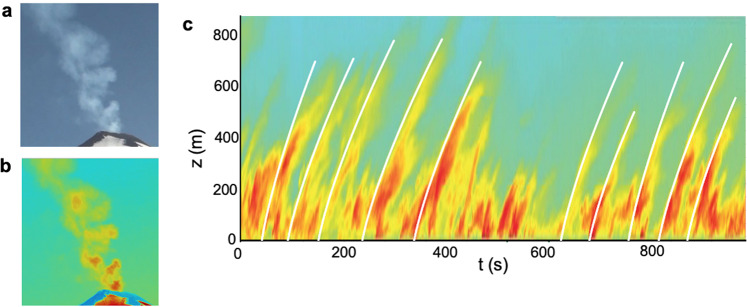
Volcanic near-vertical plume of Villarrica (2012). **a** Visible and **b** false colour images of the Villarrica plume. **c** Time series of a vertical line through the centre of the Villarrica plume. The white lines illustrating the best fit to each of the turbulent structures as they move up through the plume.

We now analyse the speed of turbulent structures in a visual video from a gas plume issuing from Villarrica Volcano in February 2012^20^. In Fig. 3 we show (a) a visible image and (b) a false colour image of the plume, and (c) we present in false colour a time series of a vertical line of pixels through the centre of the plume. This reveals a series of convective structures rising through the plume. As the volcanic gas rises through the atmosphere, the pressure and temperature of the air change. If the lower atmosphere is approximately well-mixed, then the pressure and temperature change along an adiabat. In this case, we expect the motion of the plume, driven by the buoyancy, to follow the same model as given above, provided that the pressure and density are evaluated relative to the adiabat^23,26^. This simplification should apply to the Villarrica gas plume as it rises through the first 600–800 m above the vent, and hence is not impacted by the ambient stratification.

To test the model, for each height in the image (Fig. 3c), we determine the points of maximum local gradient in light intensity with time. This leads to a series of points on the leading edge of the convective structures, and we retain the curves which extend furthest from the source. We then find the best fit line, as given by Eq. (2), to each of these sets of points on the leading edge of the convective structures. We plot these curves as white lines in Fig. 3c. Each curve leads to an estimate for *B* for each convective structure. There is no thresholding used in the image, since we have focussed on the plume and immediate surroundings in our analysis. Using these data, we estimate that the family of convective structures in the Villarrica plume are consistent with a buoyancy flux in the range *B* ≈ 930 ± 260 m^4 ^s^−3^, corresponding to an uncertainty of about 28%, using the empirical value *α* = 5.6 (see Eq. (3) above), with the best fit for the virtual origin *z*_0_ which is estimated to be 50 m below the rim of the vent. The length scales in the video have been estimated using images of the summit crater, which has a diameter 250 m^27^. We now use this buoyancy flux to estimate the gas mass flux associated with the plume.

### Vertical plumes: buoyancy flux, mass flux and gas temperature

To convert the buoyancy flux to a gas flux, we account for the flux of heat *Q*_*V*_ and the mass flux *M* of the volcanic gas in the plume. Both of these properties are conserved in the lower part of the plume as air is entrained into the plume. However, since the volcanic gas may initially be very hot, with temperature *T* + Δ*T*_*V*_ much higher than the ambient temperature *T*, then as air is mixed into the plume, and the mixture cools and the gas contracts, the buoyancy flux gradually evolves towards a constant. We now explore how this buoyancy flux depends on the source mass flux and temperature. This can be used to estimate the range of possible source mass fluxes based on the possible range of source temperatures.

To calculate the buoyancy flux, we note that as air is entrained, heat conservation requires that the mixture temperature *T* + Δ*T*_*A*_ is given in terms of the mass flux of entrained air, *M*_*A*_

4 $${Q}_{V}={M}_{V}{C}_{V}{{\Delta }}{T}_{V}=\left({M}_{V}{C}_{V}+{M}_{A}{C}_{A}\right){{\Delta }}{T}_{A}$$

where *M* is the mass flux and *C* the specific heat capacity, while the density of the mixture of volcanic gas and entrained air is

5 $$\rho =\frac{{M}_{V}+{M}_{A}}{{M}_{V}/{\rho }_{V}+{M}_{A}/{\rho }_{A}}$$

where *ρ*_*V*_ and *ρ*_*A*_ are the density of the volcanic gas and the air mixed into the plume respectively, each of which may be calculated using the ideal gas law

6 $$P=\rho RT$$

where *R* is the gas constant, which has value 287 J kg^−1^ K^−1^ for air and 461 J kg^−1^ K^−1^ for water vapour^28^. For the volcanic gas, we use the mass-averaged value of *R* based on the different volatile species, but given that water vapour normally constitutes over 95% of the mass of volcanic gas^29^, this average is approximately given by the value for water vapour. The buoyancy of the mixture is given by

7 $$g^{\prime} =g\left(\frac{{\rho }_{0}-\rho }{{\rho }_{0}}\right)$$

where *g* = 9.81 ms^−2^ is the acceleration of gravity and *ρ*_0_ is the ambient density of the air. Combining these results, we estimate that after a mass flux *M*_*A*_ of air has been entrained into the plume, the buoyancy flux, *B*, is given by

8 $$B=g^{\prime} \left(\frac{{M}_{V}+{M}_{A}}{\rho }\right)$$

**Fig. 4:**
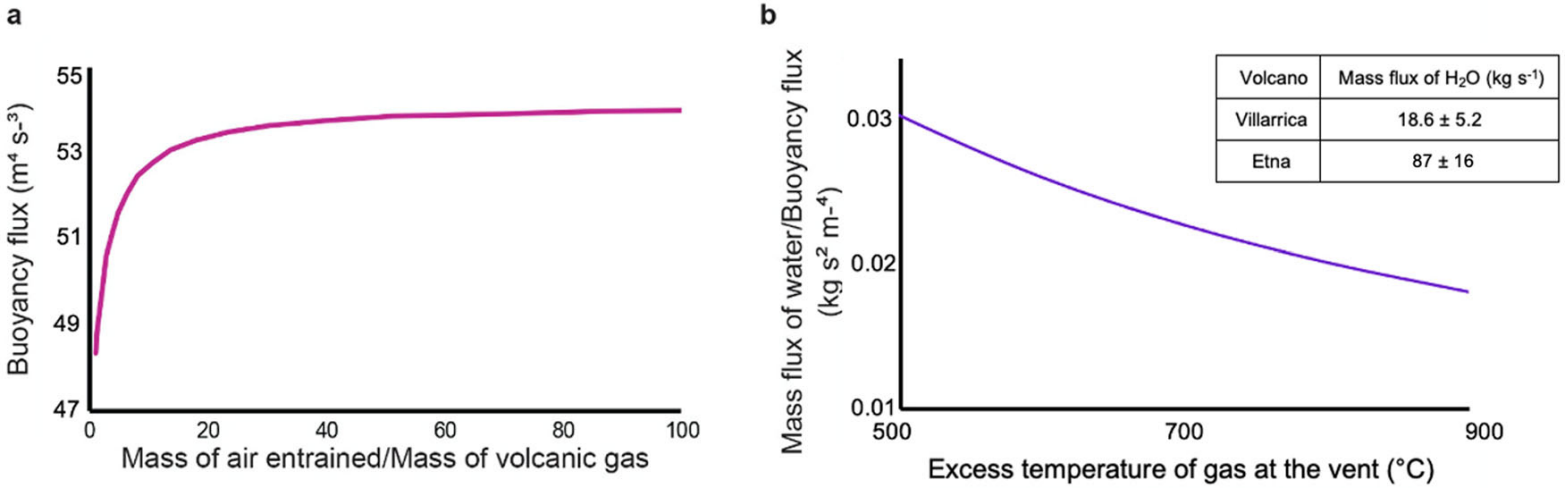
Buoyancy and mass fluxes. **a** Variation of the buoyancy flux in the mixture of air and volcanic gas as a function of the mass of air entrained into the plume, with the volcanic gas assumed to be dominantly water vapour with an initial temperature of 900 °C in excess of the ambient. **b** Variation of the mass flux of water vapour divided by the buoyancy flux as a function of the initial temperature of the volcanic gas in excess of the atmospheric temperature. The table shows our estimates for the volcanic gas mass fluxes for Villarrica Volcano and Mount Etna.

In Fig. 4a, we illustrate how the buoyancy flux *B* evolves as a function of the mass of entrained air, for a typical set of eruption conditions. There is a small increase in the buoyancy flux as the initial air is entrained, the mixture cools and the density increases. However, once the mass of entrained air is about 10 times that of the volcanic gas, the buoyancy tends to a constant.

Using the buoyancy flux estimated from the speed of convective structures rising through the plume, which corresponds to the asymptotic buoyancy flux following the entrainment of air (Fig. 4a), we can now estimate the mass flux of gas issuing from the volcano as a function of the initial temperature of the gas by combining Eqs. (4)–(8). In Fig. 4b we illustrate the variation of *M*_*V*_/*B* with the initial gas temperature. Given the buoyancy flux *B* we can then use this figure to estimate the source gas flux, *M*_*V*_. Note that in these calculations we assume the volcanic gas is primarily composed of water vapour.

For the Villarrica plume, we assume that the erupting gas has a temperature comparable to the magma; although there is no documented measurement for this eruption, magma at Villarrica has been measured to have a temperature of 1134 °C^30^, and so we assume a temperature of 1134 ± 50 °C herein. Based on Fig. 4b, this leads to an uncertainty in the conversion from buoyancy flux to H_2_O flux of 7.5%, and hence we estimate that the H_2_O flux has value 18.6 ± 6.8 kg s^−1^. Although there is no estimate of the H_2_O flux for the actual time when the video was recorded, published estimates of the SO_2_ flux at Villarrica Volcano are 1.5 kg s^−1^^17^ and 3.7 kg s^−1^^31^. Given that the molar ratio of H_2_O to SO_2_ in the gas issuing from Villarrica is between 35^31^ and 65^17^, and the molar mass ratio of H_2_O to SO_2_ is 18:64, these published SO_2_ fluxes imply a H_2_O flux of 27–36 kg  s^−1^, which is a little higher than, but of similar magnitude to the range we estimate from the plume dynamics. The main source of uncertainty in our estimate lies in the range of estimates of *B* owing to the turbulent fluctuations in the rate of ascent of the convective structures (Fig. 3c).

### Wind-blown plumes: model shape of the plume

A number of models of wind-blown plumes have been developed based on pioneering laboratory experiments^32^. Many adopt a Lagrangian approach, in which the model follows the ascent of the fluid in the plume^32–34^. These models have shown that provided the source momentum is not too large^34–37^, then over a relatively short distance after leaving the source, the flow adjusts so that the downwind speed in the plume matches the wind speed, and the subsequent motion of the plume fluid relative to the ambient is dominantly in the vertical direction owing to the buoyancy of the plume fluid. If the buoyancy flux in the plume is *B*, then the buoyancy flux per unit distance downwind is *B*/*w*, where *w* is the wind speed. Once the rise speed is smaller than the wind speed, the buoyant fluid rises in a similar fashion to a line thermal. Provided that the ambient is unstratified, we expect the local vertical mean speed, *U*, at height *z*, to be given by dimensional arguments as^22,23^

9 $$U \sim \gamma {\left(\frac{B}{w}\right)}^{\frac{1}{2}}{(z+{z}_{o})}^{-\frac{1}{2}}$$

where *γ* is an empirical constant and *z*_*o*_ is the virtual origin of the source. If we move with the fluid, then the height of the plume increases with time, *t*, as

10 $${(z+{z}_{o})}^{\frac{3}{2}}={z}_{0}^{\frac{3}{2}}+\frac{3}{2}\gamma {\left(\frac{B}{w}\right)}^{\frac{1}{2}}\left(t-{t}_{0}\right)$$

where *t*_0_ is the time of release from the source, while the distance downwind increases with time according to the relation

11 $$x={x}_{0}+w\left(t-{t}_{0}\right)$$

where *w* is the wind speed, *x* is the horizontal distance from the source, and *x*_0_ is the horizontal position of virtual origin which accounts for the acceleration of the flow to the wind speed. Combining Eqs. (10) and (11), we expect that far downstream, the plume will follow the approximate trajectory

12 $${(z+{z}_{0})}^{\frac{3}{2}}={z}_{0}^{\frac{3}{2}}+\frac{3}{2}\gamma {\left(\frac{B}{{w}^{3}}\right)}^{\frac{1}{2}}(x-{x}_{0})$$

This model coincides with the predictions of the Lagrangian plume models in the region far downwind^32–34^ and has been shown to be consistent with small-scale laboratory experiments^34^. In the next section, we report some new experiments of such plumes, in which we examine the ensemble average of a video recording of the plumes for several values of the wind speed, and thereby estimate the value of *k*.

### Wind-blown plumes: laboratory experiments

**Fig. 5:**
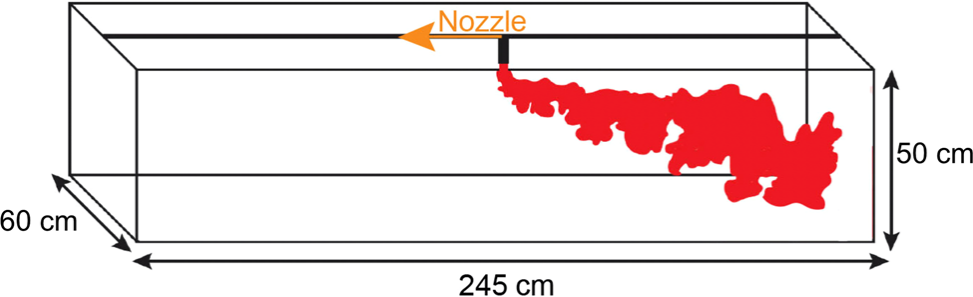
Schematic illustration of the wind-blown plume in the experimental tank. A nozzle is located at the top of the tank and moves at a constant speed from the right to the left side of the tank during an experiment. Six experiments were carried out, with nozzle speeds of 0.104, 0.092, 0.087, 0.075, 0.056 and 0.037 ms^−1^.

We have carried out a series of experiments in a flume tank filled with water; the experimental tank was 245 cm long, 60 cm wide and 50 cm deep (see Fig. 5). A continuous source of saline water solution (6.2 wt.% NaCl) was supplied to the tank; this source was moved along the tank at six different constant speeds of 0.104, 0.092, 0.087, 0.075, 0.056 and 0.037 ms^−1^. The source volume flux of 5.27 cm^3^ s^−1^ was supplied using a Watson Marlow 520 N peristaltic pump. The tank was backlit with a uniform light sheet and the experiments were recorded using a Nikon D5300 camera with a frame rate of 50 Hz.

**Fig. 6:**
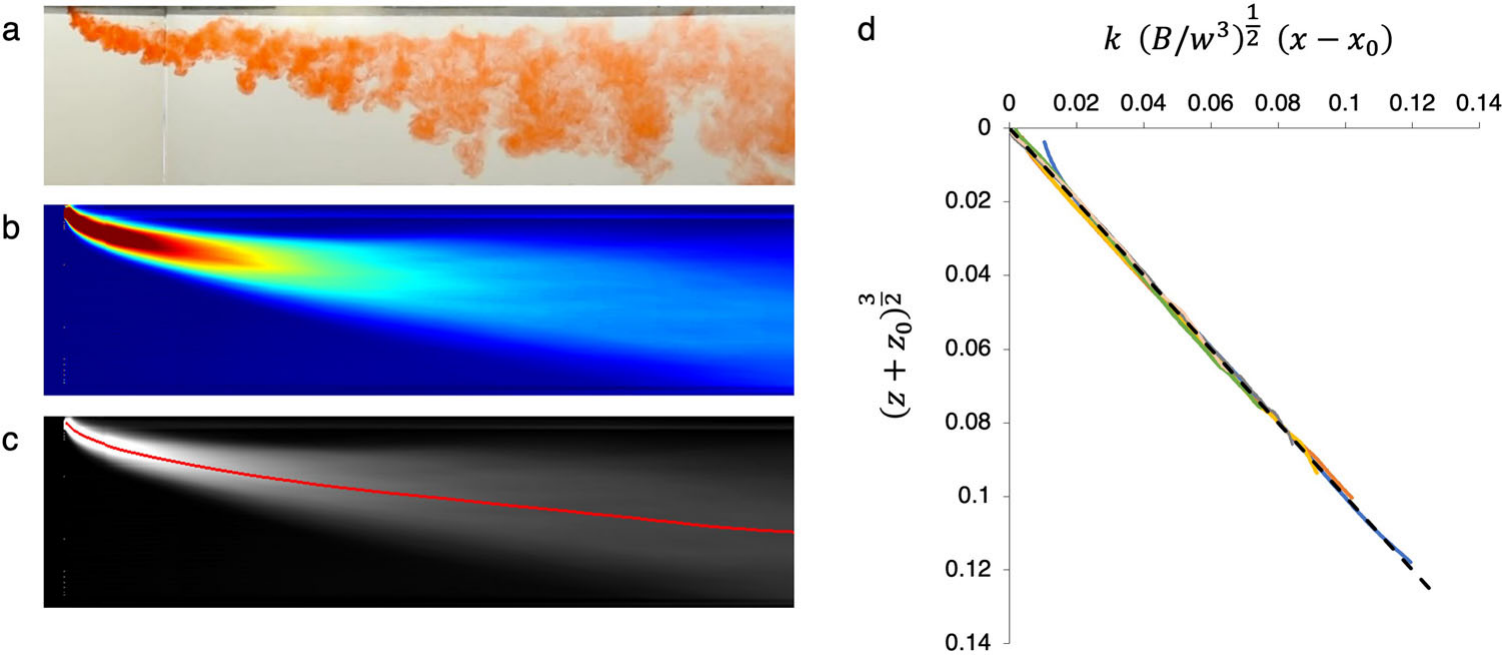
Laboratory-scale wind-blown plumes. **a** Experimental picture of a wind-blown plume illustrating the highly turbulent nature of the flow. **b** Time average of the images of the plume, in false colour, showing the evolution of the ensemble average of the dye concentration in the plume. **c** Image showing the centre of mass of the plume at each point downwind (red curve), based on the vertical distribution of the mass of dyed fluid at that point. **d** Comparison of the scaling law for the shape of the plume, plotted as ${\left(z+{z}_{0}\right)}^{3/2}$ as a function of $k{\left(B/{w}^{3}\right)}^{1/2}\left(x-{x}_{0}\right)$. The results of four experiments (coloured lines) are compared to the prediction of the model (black line).

In Fig. 6a we show an image of the plume in the experimental tank as it spreads downwind, and in Fig. 6b we show the average plume shape, obtained by averaging the frames captured over 38 s during the experiment. This time-averaged frame is shown in false colours to emphasize the concentration variations through the plume. Using this time-averaged image, for each vertical line through the plume, we have calculated the position of the centre of mass of the plume (Fig. 6c). For each of six experiments with different model wind speeds, *w*, we compare Eq. (12) with the experimental data for the centreline as a function of distance from the source. We estimate the values of *x*_0_, *z*_0_ and *k* for which the difference between $k{\left(B/{w}^{3}\right)}^{1/2}\left(x-{x}_{0}\right)$ (horizontal axis in Fig. 6d) and ${\left(z+{z}_{0}\right)}^{3/2}$ (vertical axis in Fig. 6d) is minimised. We find that for the different values of *w* used in the experiments, the corrections used to estimate the virtual origin of the plume are small, with *x*_0_/*L* = 0.023 ± 0.018 relative to the horizontal length of the plume in the tank, *L*, and *z*_0_/*H* = 0.006 ± 0.015 relative to the vertical height of the plume, *H*. We also find that *k* = 0.86 ± 0.03.

### Wind-blown plumes: Mount Etna (2015)

**Fig. 7:**
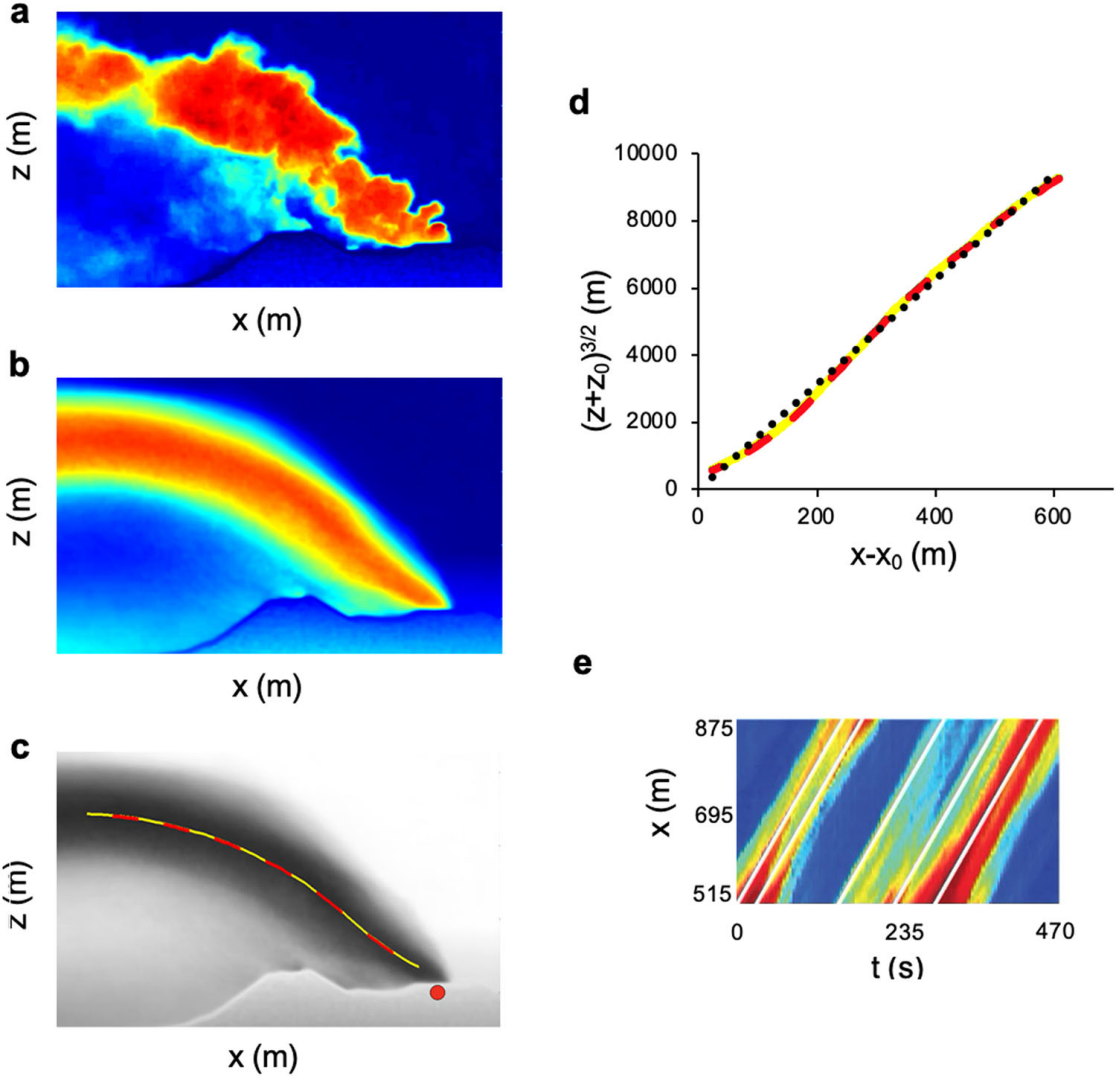
Volcanic wind-blown plume at Mount Etna (2015). **a** False colour image of the plume obtained from the UV movie. **b** Image of the overall shape of the plume in false colour. **c** The red and yellow dashed lines illustrate the vertical height of the centre of mass in the plume, as estimated directly from the data (red) and also from fitting the pixel value histogram to a Gaussian distribution at each point along the plume, and mapping the locus of the centre of these Gaussian distributions (yellow). The red dot illustrates the approximate position of the virtual source of the plume. **d** Comparison of the estimates of height of the centre of mass of the plume as a function of distance downwind (red and yellow dashed curves) with the model curve (Eq. (10)) shown with black dots. **e** Image of the vertical average of the pixel values at each downwind point in the plume, as a function of time. The white lines indicate the turbulent structures in the flow. We used these straight lines to estimate the average wind speed *w* = 2.3 ± 0.10 ms^−1^, corresponding to an error of about 4%.

We have analysed a movie of the gas plume venting from Etna’s north east crater (NEC) recorded in the UV range of light in September 2015. In Fig. 7a, we use false colours to show an instantaneous image of the plume during the eruption, with the colour in each pixel based on the pixel light intensity. In Fig. 7b, we present the time-averaged shape of the plume. The histogram of pixel values in this averaged frame is very similar to that for the average of the laboratory experiment, and so we follow the approach used to describe the experimental plume. We use the averaged image to estimate the vertical centre of mass of the plume as function of downstream distance from the source (Fig. 7c). In assessing the sensitivity of the location of the centre of mass to the distribution of pixel values, we calculate the centre of mass directly from the data (red line in Fig. 7c), and we also fit a Gaussian distribution to the data, to try to assess the centre of mass (yellow dashed line in Fig. 7c). In Fig. 7d, we compare the two estimates of the centre of mass and see that these are indistinguishable. These lines are also compared with the model equation for the shape of the plume (dotted line), and find there is reasonable agreement, provided that

13 $$\frac{{B}^{\frac{1}{3}}}{w}=6.8\pm 0.3$$

corresponding to an error of about 4%. In order to constrain the value of the wind speed *w*, we have estimated *w* by plotting a time series of horizontal lines through the plume, as illustrated in Fig. 7e. Here, we see a series of lines that correspond to turbulent structures in the flow, which move with the model wind speed. From the slope of the lines, we estimate that *w* = 2.3 ms^−1^. We note that when we applied the same technique for estimating *w* in the laboratory plume, we recovered the speed of the source within an error of <1%. Based on this model, we predict that the buoyancy flux *B* at Mount Etna lies in the range of 3925 ± 428 m^4^ s^−3^, corresponding to an uncertainty of about 11%.

We now use the calibration curve in Fig. 4b to convert this to a mass flux. For this conversion we assume that the initial temperature of the gas equals that of the magma; although there was no measurement of the magma temperature when the video was taken, the temperature of magma at Etna has a typical value of 1080 °C^38^, and so we assume it lies in the range 1080 ± 50 °C. This leads to an uncertainty in the conversion from buoyancy flux to H_2_O flux of about 7.5%, and using the buoyancy flux estimated above, we predict that the volcanic gas flux, *M*_*V*_, lies in the range 87 ± 16 kg s^−1^. Here, the main source of uncertainty lies in the use of plume theory to model the shape of the plume, combined with the estimate of the local wind speed experienced by the plume. Although there is no estimate of the H_2_O flux for the actual time when the video was recorded, the SO_2_ fluxes reported in previous studies from Etna are 18 kg s^−1^^15^. From this, we estimate the H_2_O flux to be 186 kg s^−1^ by using the measured molar H_2_O/SO_2_ ratio from the NEC crater of 36^39^ and combining this with the molar mass ratio of 18:64 for H_2_O to SO_2_, which is of comparable size, although a little larger than our estimate. The length scale in the images was estimated by reference to the length scale of the northeast crater (NEC), which has a diameter of about 200 m^40^.

## Discussion

Turbulent buoyant plumes develop convective eddy-type structures, which are carried upwards in the plume, and their speed provides information about the underlying buoyancy flux of the plume. The ascent speed of the turbulent structures in the vertical volcanic gas plume at Villarrica Volcano analysed in this paper is consistent with this model and provides an estimate of the buoyancy flux. We have then developed a simple model to convert this to a mass flux of gas issuing from the volcano. We have also considered the shape of a wind-blown gas plume at Mount Etna, and shown this is consistent with the classical models and experiments of the shape of a wind-blown turbulent buoyant plume far downwind. By comparing the shape of the Etna plume with the model, we estimate the buoyancy flux of that plume, and again using our model, we convert this to the source gas mass flux. The gas flux values we find are comparable to the typical estimates of gas fluxes at these volcanoes derived from spectroscopy combined with electrochemical sensors.

This study paves the way to using high-quality video images of gas plumes to quantify total volatile mass fluxes. If the plume gas composition is known via the measurement of the chemical gas composition at the crater rim with sensor packages or FTIR, the flux of individual species may be estimated. This new method gets around the difficulties of estimating fluxes using spectroscopy when plumes are vertical and not in an ideal configuration for traverses. Furthermore, this method could also be used to estimate fluxes in sulfur-poor plumes, which are difficult to characterise by spectroscopy (e.g., plumes at hydrothermal systems at Yellowstone or White Island).

There is also some uncertainty associated with the use of plume theory to derive the estimates of the buoyancy flux, especially since the trajectories of the plumes depend on the fractional powers of the source buoyancy flux. For the vertical plume the turbulent fluctuations lead to an uncertainty of about 28%, while for the horizontal plume they combine to give an uncertainty of about 24%. Measurement of the magma temperature at the surface would remove some uncertainty in the conversion from buoyancy flux to mass flux. Nonetheless, the approach provides an independent estimate of the gas flux and hence complements the other approaches mentioned in the introduction.

## Data Availability

The data sets generated during and/or analysed during the current study are available from the corresponding author on reasonable request.
